# Etiology and patterns of mandibular fractures in cats

**DOI:** 10.3389/fvets.2025.1613902

**Published:** 2025-06-18

**Authors:** Ana C. Castejón-González, Darko Stefanovski, Alexander M. Reiter

**Affiliations:** ^1^Department of Clinical Sciences and Advanced Medicine, School of Veterinary Medicine, University of Pennsylvania, Philadelphia, PA, United States; ^2^Department of Clinical Studies-New Bolton Center, School of Veterinary Medicine, University of Pennsylvania, Philadelphia, PA, United States; ^3^Tierzahnzentrum München, Munich, Germany

**Keywords:** mandibular fracture, cats, 3D-printing, maxillofacial trauma, animal altercation, fracture patterns

## Abstract

**Introduction:**

Mandibular fractures resulting from maxillofacial trauma often require surgical intervention to promote proper bone healing. Understanding the etiology and patterns of mandibular fractures is crucial for selecting appropriate surgical treatment options. The objectives of this study were (1) to examine the etiology and location of mandibular fractures at and distal to the mandibular canine tooth and (2) to identify patterns and risk factors associated with these fractures in client-owned cats.

**Methods:**

Medical records and computed tomography (CT) scans of cats with at least one mandibular fracture located at or distal to the mandibular canine tooth were reviewed. The CT images of mandibles with ramus fractures were segmented and reconstructed into 3D models using the Mimics Innovation Suite (Materialise, Leuven, Belgium). These models were then printed in white or clear resin using an SLA 3D printer (Formlabs©) to identify fracture patterns.

**Results:**

A total of 38 cats with 62 mandibular fractures were included in the study. The most common fracture location was the mandibular ramus (51.6%, excluding the condylar process), followed by the condylar process (33.9%). Fractures were often severely displaced and fragmented. The evaluation of the 3D-printed models identified three main patterns, which accounted for 75% of the fractures in the mandibular ramus. Fracture etiology was significantly associated with the pattern type (*p* = 0.028). Animal altercations were 9.3 times more likely to cause a pattern A fracture than an unknown cause.

**Discussion:**

3D printing was useful for visualizing and describing the patterns of mandibular fractures in cats. Pattern A fractures were most commonly associated with animal altercations.

## 1 Introduction

Recent studies on craniomaxillofacial trauma in cats have shown that mandibular fractures are common. Between 72 and 86% of cats with maxillofacial trauma experience mandibular fractures (1, 2). The majority of fractures occur in the mandibular ramus (not including the condylar and angular processes) and the condylar process, often as a result of vehicular accidents (1, 2).

Treatment selection depends, among other factors, on the location of the fracture (3, 4). Fractures of the mandibular body can successfully be repaired with wire-reinforced interdental bis-acryl composite splints (4, 5). Management of caudal mandibular fractures using different forms of maxillomandibular fixation can also yield favorable results. Techniques include the bi-gnathic encircling and retainer device (BEARD), labial buttons with sutures, elastic chains, and composite splints placed between the maxillary and mandibular canine teeth (3, 6–8). However, these techniques do not prevent the movement of the caudal bone fragments caused by masticatory muscle contractions, can interfere with the patient’s ability to eat, and may pose a fatal risk in cases of vomiting, regurgitation, or aspiration of stomach contents (3). In addition, they may not be sufficient to achieve bone healing because these techniques do not provide approximation and stabilization of displaced fracture fragments. Open reduction and internal fixation (ORIF) with titanium implants (such as locking miniplates and ramus anatomical plates) is preferred because it provides strong stabilization of fracture fragments and quick return to normal masticatory function (9–11). However, plate placement can be challenging due to the small size of bone fragments, the thin bone in the masseteric fossa, and the curved shape of the ramus. Furthermore, malocclusion is a common complication of maxillofacial trauma (12, 13). Even slight malignment of the fragments can cause malpositioned teeth and possible tooth-to-tooth, tooth-to-palate, or tooth-to-soft tissue contact.

It is important to understand the geometry of mandibular fractures and the risk factors associated with specific fracture patterns. This knowledge aids in surgical planning and provides information regarding possible treatment outcomes. In addition, understanding fracture patterns is crucial for the development of new techniques to treat challenging fractures. Mapping of mandibular angle fractures in cats showed two main patterns. The first pattern begins at the junction of the mandibular ramus and body, extending toward the angular process in a relatively straight line. The second pattern follows a sigmoid shape around the mandibular foramen before curving toward the angular process (14). The study found no associated risk factors (14).

The objectives of the present study were (1) to characterize mandibular fractures at and distal to the mandibular canine tooth, (2) to identify the associated risk factors, and (3) to determine specific fracture patterns in the mandibular ramus. We hypothesized that specific patterns are common in the mandibular ramus and that those patterns are dependent on fracture etiology.

## 2 Materials and methods

### 2.1 Case selection

Medical records and computed tomography (CT) scans of cats diagnosed with acute mandibular fractures and presented to a referral center between 2008 and 2024 were evaluated. Patients with at least one mandibular fracture in the mandibular body (between the canine and first molar teeth) or ramus (distal to the first molar tooth) were included. The exclusion criteria were symphyseal separation or parasymphyseal fracture as the sole injury in the mandible and evidence of non-union or remodeling of bone fragments indicative of chronic injuries. Cats with fractures secondary to oral neoplasia were also excluded. All cats underwent a conventional CT scan at the time of presentation. Only bone algorithm images with a slice thickness of ≤1 mm were analyzed.

### 2.2 Medical records and CT scan review

From the medical records, signalment and cause of the fracture were retrieved. The CT images were analyzed to determine the fracture location, number of fractures, displacement, fragmentation, whether fractures were unilateral (affecting one side of the mandible) or bilateral (involving both mandibles), and symmetry. Symmetry was defined as the presence of a mandibular fracture in the same area (the body, ramus, or condylar process) of the opposite mandible. Symphyseal separation or parasymphyseal fractures were not taken into consideration for the evaluation of laterality and symmetry. The presence of maxillary fractures was also recorded.

Due to treatment implications, fractures were classified based on their location as either mandibular body fractures (between the canine and first molar teeth) or mandibular ramus fractures (distal to the first molar). Although the condylar process is anatomically part of the ramus, for the purpose of this study, fractures of the condylar process were excluded from the ramus category, as internal fixation of condylar fractures is, to our knowledge, not possible. Instead, fractures of the condylar process were registered separately from mandibular ramus fractures. Symphyseal separation or parasymphyseal fractures were considered a single entity. Displacement was classified as no displacement (1), minimal displacement (more than 50% overlap between fragments) (2), and severe displacement (less than 50% overlap between fragments) (3). Fragmentation was classified as incomplete (1), simple (2), and comminuted (3) (three or more fragments).

### 2.3 3D printing

DICOM files from the patients with ramus fractures were uploaded into virtual-aided surgery software (Mimics Innovation Suite, Materialise, Leuven, Belgium) to create a 1:1 3D model of each affected mandible. A segmentation tool (Materialise Mimics) was used to create separate parts for each fracture fragment. When the fracture was comminuted into small fragments, these fragments were segmented and attached to one main fragment to avoid missing them during the printing process. The files corresponding to each part were exported to 3matic (Materialise 3-matic) to finalize the modeling. The 3D models (. STL files) were printed using an SLA printer (Form 3B+, Formlabs©) in white or clear resin with adaptive layer thickness. The printed models were used to evaluate the geometry of each ramus fracture.

### 2.4 Statistical analysis

The descriptive analysis included the computation of means and SD. Categorical variables were reported as frequency counts and percentages. Spearman’s rank correlation coefficient was used to identify significant associations between the independent variables and fracture location and fracture pattern. The independent variables included gender, breed, age, weight, cause of trauma, additional maxillary fractures, whether the fracture was unilateral or bilateral, symmetry, displacement, and fragmentation.

For all pairwise associations, the *p*-value and Spearman’s rho coefficient were reported. The subset of independent variables associated with each outcome was further analyzed using a stepwise backward multivariate logistic regression model to identify the subset of variables significantly associated with the outcome. All analyses were conducted using Stata 18MP (StataCorp, College Station Tx) with two-sided hypothesis tests, and a *p*-value of <0.05 was considered statistically significant.

## 3 Results

### 3.1 Fractures

A total of 38 cats were included in the study. The mean age was 6.11 ± 4.8 years, and the mean body weight was 4.7 ± 1.5 kg. A total of 26 (68.4%) cats were male, of which five were intact, and 12 cats (32.6%) were female, of which two were intact. There were 35 domestic Shorthair cats, one domestic Longhair cat, one Turkish Angora cat, and one Scottish Fold cat. 26 (68.2%) cats had other maxillofacial fractures in addition to mandibular fractures, and 27 (71.1%) cats had symphyseal separation or parasymphyseal fractures. Only three (7.9%) cats had mandibular fractures without any accompanying maxillofacial fractures or symphyseal separation/parasymphyseal fractures.

**Figure 1:**
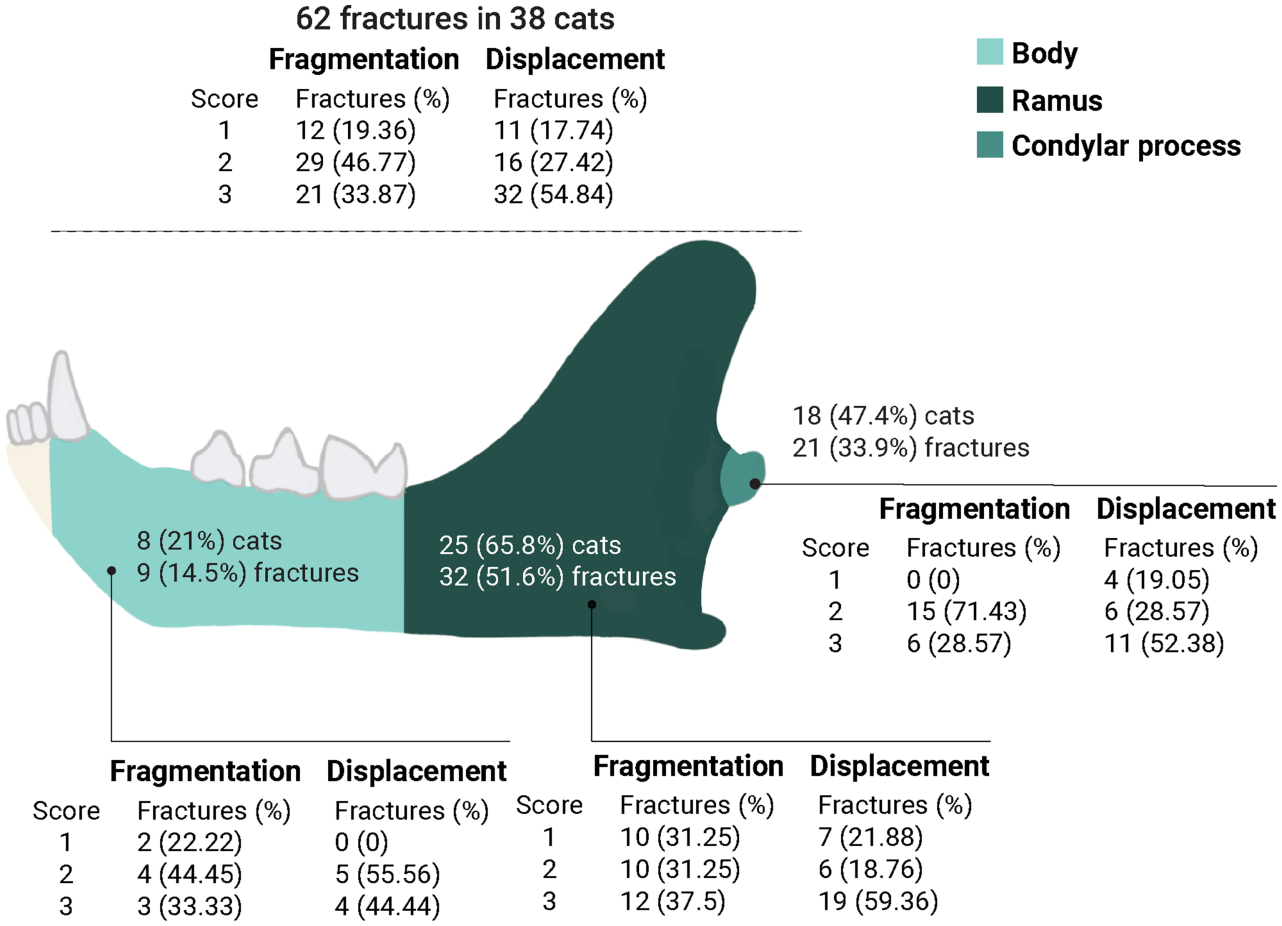
Fragmentation and displacement of the 62 fractures diagnosed in 38 cats. Fragmentation scores: (1) incomplete fracture, (2) simple fracture, and (3) comminuted fracture (3 or more fragments). Displacement scores: (1) no displacement, (2) minimally displaced fragments (>50% overlap between fragments), and (3) severely displaced fragments (<50% overlap between fragments).

A total of 62 mandibular fractures were identified, of which 32 (51.6%) were located in the mandibular ramus, 21 (33.9%) in the condylar process, and nine (14.5%) in the mandibular body (Figure 1). Animal altercations (bite injuries) were the most common cause of mandibular fractures (42.1% cats; 40.3% fractures), followed by an unknown cause (29.9% cats; 29% fractures), hit-by-car incidents (15.8% cats; 14.5% fractures), injuries resulting from falling from a height (12.5% cats; 14.5% fractures), and ballistic injury (3.1% cats; 1.6% fractures). There were 1.6 ± 0.7 mandibular fractures per cat.

Half of the cats (*n* = 19) had bilateral fractures, of which 42.1% (*n* = 8) were symmetric (affecting the same location in both mandibles). There was a significant negative correlation (*p* = 0.0066) between body weight and bilateral mandibular fractures (Table 1). Smaller cats were significantly associated with bilateral fractures (*p* = 0.004) in the logistic regression model.

**Table 1:** Correlation between mandibular ramus fracture patterns and cause (*p* < 0.05, Spearman rank test).

Variable 1	Variable 2	*p*-value	Rho^*^
Fracture pattern	Cause	0.010	0.4113
Fracture pattern	Maxillary fracture	0.002	0.3853
Age	Unilateral/bilateral fractures	0.006	−0.3435

^*^Rho is the Spearman’s correlation coefficient. It varies from −1 to 1, where 1 indicates high correlation, −1 indicates high inverse correlation, and 0 indicates no correlation.

The majority of the fractures (*n* = 34; 54.84%) were severely displaced (score 3) and had fragmentation scores of 2 or 3 (*n* = 50; 80.64%) (Figure 1).

### 3.2 Fracture patterns

**Figure 2:**
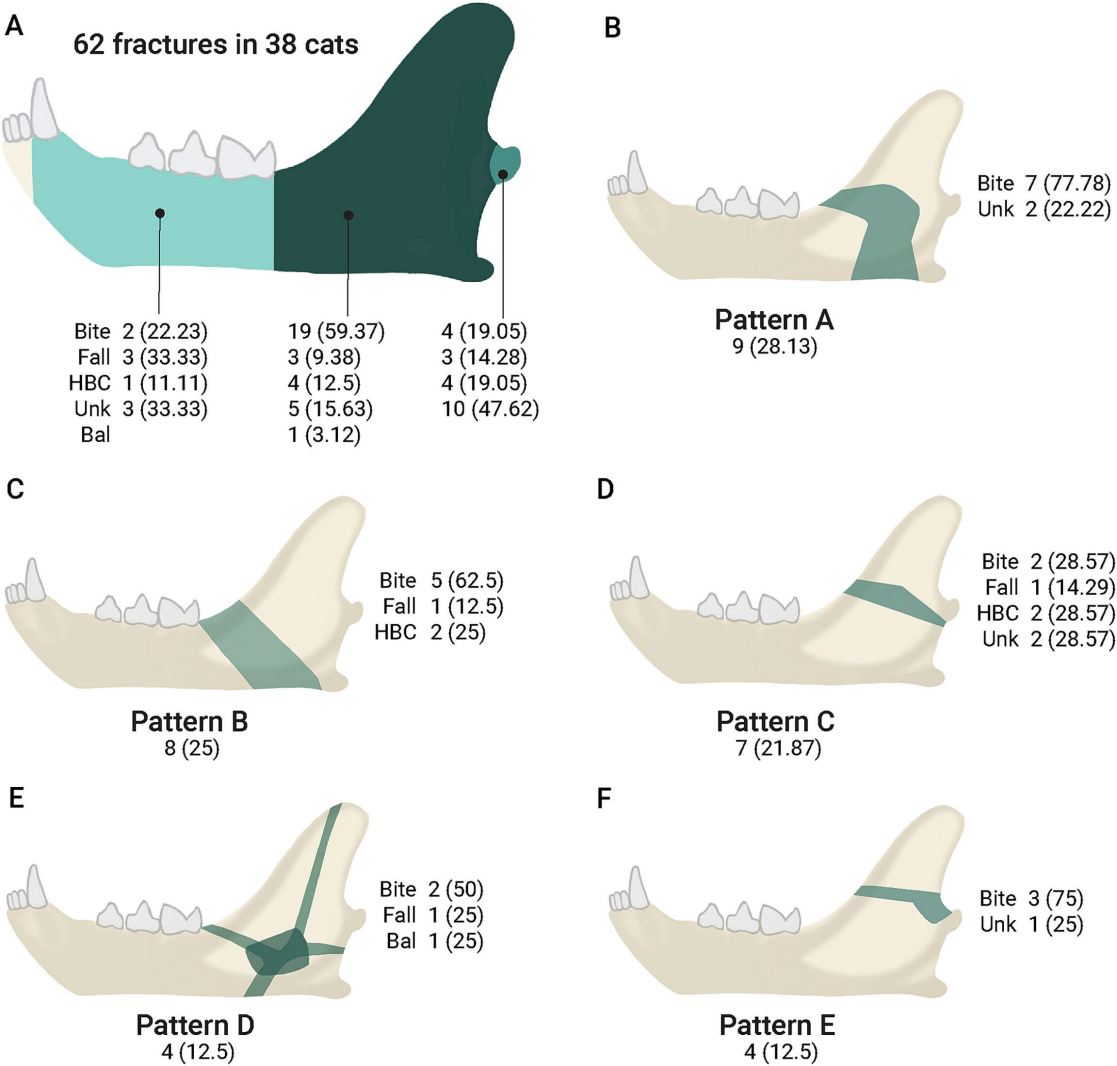
Fracture etiology by location (mandibular body, mandibular ramus, and condylar process) and fracture patterns. **(A)** Fractures (percentage) in the body, ramus, and condylar process. **(B–F)** Ramus fracture patterns, showing the number (percentage) of fractures within each pattern. The green shadow indicates the location and main shape of the fracture (patterns A to E). Bal = ballistic injury; HBC = hit by a car; Unk = unknown etiology.

**Figure 3:**
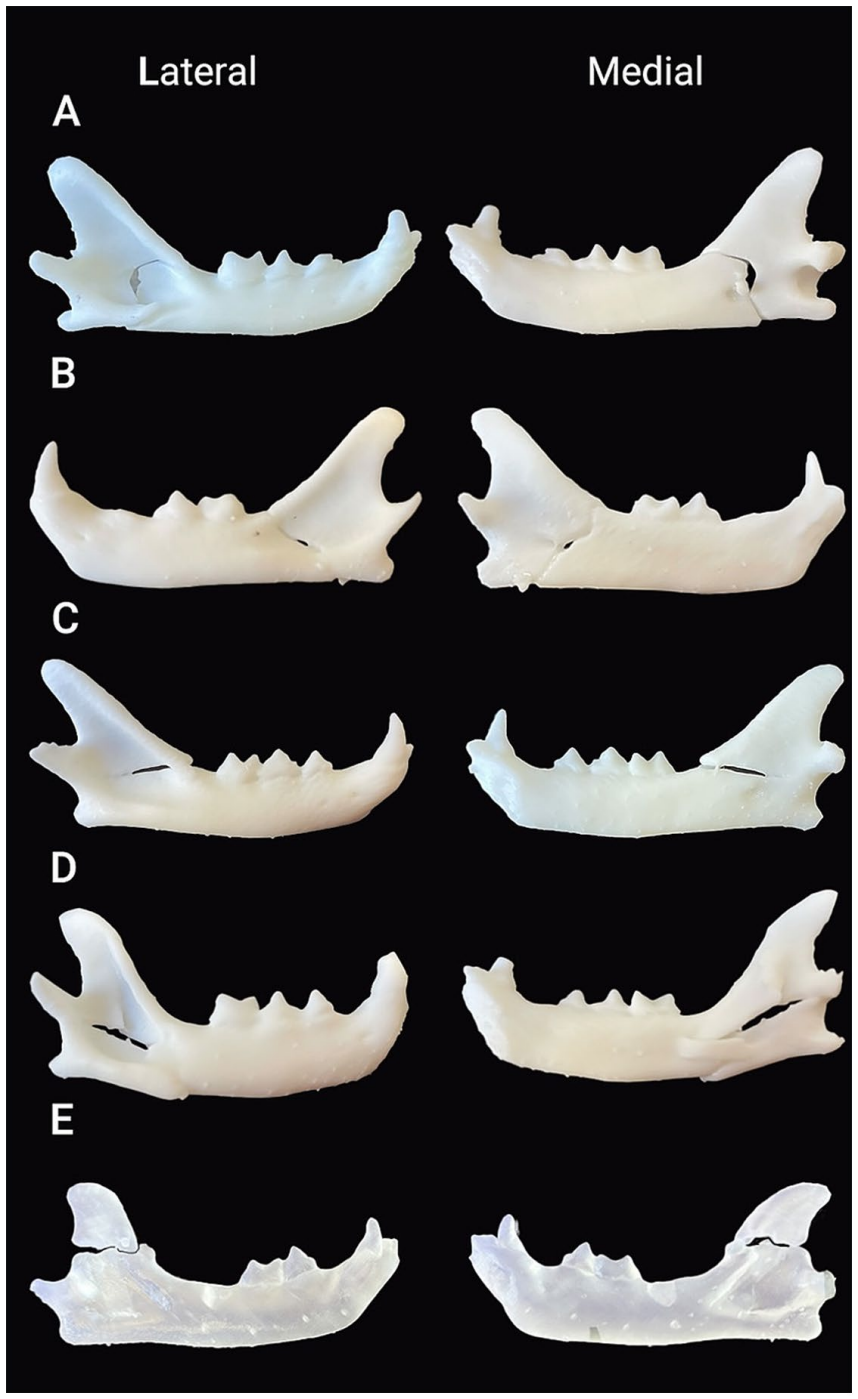
Lateral and medial views of the 3D-printed models of the mandibles with ramus fractures in white **(A–D)** and clear **(E)** resin. **(A)** Pattern A fracture: A fracture that started dorsally at the transition of the mandibular body and ramus, continued caudally into the masseteric fossa and, before reaching the temporomandibular joint, it curved ventrally towards the mandibular foramen and the ventral mandibular cortex rostral to the angular process. **(B)** Pattern B fracture: An oblique fracture that started dorsally immediately distal to the first molar tooth or near the transition between the body and ramus, continued caudally toward the mandibular foramen, and ended in the ventral mandibular cortex rostral to the angular process. **(C)** Pattern C fracture: A fracture that started near the transition between the body and ramus, extended caudally in a relatively straight line, and ended immediately ventral to the condylar process. **(D)** Pattern D fracture: An explosive comminuted fracture centered in the masseteric fossa, with multiple fracture lines extending toward its periphery in multiple directions. **(E)** Pattern E: A fracture line running from the rostral aspect of the coronoid process to its caudal aspect dorsal to the condylar process.

The evaluation of the ramus fractures showed five different patterns, with three of them accounting for 75% of the fractures in that location. The most common pattern (*n* = 9 fractures; 28.13%) was a fracture that started dorsally at the transition of the mandibular body and ramus, continued caudally into the masseteric fossa and, before reaching the temporomandibular joint, it curved ventrally towards the mandibular foramen and the ventral mandibular cortex rostral to the angular process (pattern A). A caudal oblique fracture starting immediately distal to the first molar tooth or near the transition between the body and ramus, continuing caudally toward the mandibular foramen, and ending in the ventral mandibular cortex rostral to the angular process (pattern B) was the second most common pattern (*n* = 8; 25%). The third most common pattern (*n* = 7; 21.87%) was a fracture starting near the transition between the body and ramus, extending caudally in a relatively straight line, and ending immediately ventral to the condylar process (pattern C). All pattern C fractures were incomplete. The last two patterns included an explosive comminuted fracture that was centered in the masseteric fossa and extended in multiple directions toward its periphery (*n* = 4; 12.5%) (pattern D) and a fracture of the coronoid process starting at the rostral aspect of the ramus and ending dorsal to the condylar process (*n* = 4; 12.5%) (pattern E). One pattern E fracture extended ventrally into the masseteric fossa, continued toward the condylar process, then curved dorsally at its caudal aspect, ending dorsal to the condylar process (Figures 2, 3). All ramus fractures were oblique in lateromedial or mediolateral orientation. The cats with bilateral ramus fractures (*n* = 6) exhibited different patterns in the right and left mandibles. One mandible had a pattern A (*n* = 4) or B (*n* = 2) fracture, while the opposite mandible had a pattern B (*n* = 1), C (*n* = 2), D (*n* = 2), or E (*n* = 1) fracture. Four of these cats had an altercation with a dog. The cause of the fractures in the remaining two cats was unknown.

The fracture pattern was significantly associated with etiology (*p* = 0.010) and the presence of other maxillofacial fractures (*p* = 0.002). In the multimodal regression analysis, only the fracture pattern was associated with etiology (*p* = 0.028). Compared to the fractures of unknown cause, an animal altercation was 9.3 times more likely to cause a pattern A fracture.

## 4 Discussion

The results of this study indicate specific patterns of mandibular fractures in cats and their association with the cause of the traumatic injury. The frequency and location of mandibular fractures have been reported in two other recent studies (1, 2). Our results showed similar frequencies despite differences in etiology. While vehicular accidents were reported as the most common cause of mandibular fractures in previous studies (1, 2), animal altercations were found to be the most common cause of mandibular fractures in the present study. The cause itself may not strongly influence the location of the fracture; however, it may influence the specific geometry of the fracture in the mandibular ramus.

Bilateral fractures occur more frequently in smaller cats. This can be directly related to the ability of dogs to grab the entire head of smaller cats during altercations or to the fact that the mandibles of smaller patients do not dissipate forces as efficiently as those of larger patients, potentially resulting in fractures on both sides.

As recently described (14), the majority of mandibular fractures in cats are fragmented (scores 2–3) and severely displaced (score 3). This finding has important clinical implications. Highly fragmented and severely displaced fractures are unlikely to heal through bone formation using non-invasive or minimally invasive techniques, as the fragments cannot be properly aligned to obtain bone approximation and stabilization. This is unlikely to happen with MMF. The masticatory muscles pull the caudal fragments dorsally and medially and continue to cause motion of the fragments despite the inability to open and close the mouth. Therefore, internal fixation with plates (i.e., miniplates, ramus anatomical plates, or meshes) that allow better alignment with less morbidity and a rapid return to normal masticatory function is preferred (9–11, 15). The intraosseous wiring technique may be suitable when the fracture can be anatomically reducible and interfragmentary compression can be successfully achieved (16).

Incomplete fractures (19.36%) generally do not require additional stabilization; however, they may have important clinical consequences as they exhibit some degree of displacement due to angulation and affect the alignment of the bone, causing malocclusion or stress in the temporomandibular joint.

The evaluation of the CT scans and 3D-printed models revealed that, despite appearing to consist of only two large bone fragments, there were also small fragments present in the masseteric fossa. If the dorsal and ventral borders of the mandible are not affected by comminution, intraosseous wiring may be sufficient to achieve perfect reduction and compression of the fragments along these borders, despite leaving a small defect in the masseteric fossa (16). However, achieving interfragmentary compression with intraosseous wiring may be difficult or impossible in cases where the fracture line is also oblique in the lateromedial direction. Therefore, tightening the intraosseous wires is likely to cause sliding and overlapping of the fragments, which could result in malunion and malocclusion.

The 3D-printed models allowed better manipulation and visual understanding of the fractures. Five fracture patterns were identified in the mandibular ramus, three of which accounted for 75% of the fractures in this location. Similar fracture lines have been described in two recent publications (11, 14). The fracture starts dorsally at or near the transition between the body and ramus of the mandible (patterns A, B, and C). The point of application and direction of external forces, head movement to avoid injury, contraction and insertion of the masticatory muscles, and the thin bone in the masseteric fossa ultimately influence the propagation of the fracture, resulting in a variety of specific fracture lines.

Pattern A fractures were 9.3 times more likely to result from an animal altercation than from an unknown cause. The unknown cause was the second most frequent cause of injury, and it may include a combination of factors such as being hit by a car, falling from a height, or other types of trauma, including an animal altercation.

Although it is impossible to know with certainty how the fractures occurred, we suggest possible scenarios for common fracture patterns. Pattern A fractures can be explained by the application of force to the rostral part of the mandible, creating a bending moment with further rotation due to struggle during the altercation, compounded by the laxity of the mandibular symphysis and forces from the masticatory muscles. Pattern B and C fractures may occur with minimal or no rotational forces. Pattern D fractures were present in four cats, one of which had sustained a ballistic injury. This fracture pattern can also result from bite injuries with canine teeth pressing directly over the masseteric fossa or from high-energy trauma (such as being hit by a car or a fall) concentrating forces in the same area.

As recently reported, an interesting finding was that bilateral symmetric ramus fractures always exhibited a different pattern (14). We identified pattern A or B in one mandible, while the opposite mandible showed greater pattern variability, with patterns ranging from B to E. Based on the cause of these cases, it is suggested that bilateral and symmetric fractures occur more frequently due to animal altercations.

3D-printed models of patients have been used for education, training, and treatment planning in oral and maxillofacial surgery in small animals for over 10 years (17–20). The main maxillofacial applications of virtually aided surgery in small animal patients include mandibular and maxillary tumor resections, gap arthroplasty of the temporomandibular joint, and mandibular bone repair and reconstruction (e.g., determining the length of plates, pre-bending surgical implants, and determining the volume of grafts). Instead of virtual models, printed 3D models, were used in the present study for the evaluation of the fracture patterns to manipulate and reduce the fragments physically and provide a realistic representation of the fractures.

3D-printed models are a useful tool for understanding fracture patterns (21, 22). The authors did not determine whether the evaluation of the fracture patterns using physical models versus virtual models alone was different or if any of the models was better in obtaining more clinically relevant information. However, subjectively, physical manipulation of the exact replicas of the injured mandibles provide a deeper understanding of the thickness of the bone at various locations, the size of the fragments, the possibility of reduction, and the direction of forces. These factors influence the choice of treatment and the selection of implants for each fixation. In human medicine, the use of 3D-printed models to prebend plates has been shown to reduce surgical time and costs (23). However, surgical planning of an acute fracture with a 3D model involves two different anesthetic sessions in veterinary patients: the first anesthetic session for obtaining the CT scan needed to create the diagnostic images and print the model and the second session for performing the fracture repair after the 3D model has been printed. Whether this protocol has better patient outcomes is unknown, but it is unlikely to reduce overall costs for the client.

The present study has two main limitations. The evaluation of the CT images segmentation, and 3D modeling were all performed by the same investigator, which might have introduced some bias and affected the results. However, each CT was reviewed at least three times. Another limitation is that there were many cats with an unknown cause of injury. Although it is likely that trauma in this group included vehicular accidents, falls, or other high-energy impacts, it cannot be ruled out that some mandibular fractures may also have resulted from animal altercations. The fracture patterns in the mandibular body were not analyzed for several reasons: (1) the number of cases was limited, (2) these patterns have already been reported by the authors, and (3) management of mandibular body fractures using wire-reinforced interdental bis-acryl composite splints is generally less challenging than treatment of fractures distal to the first molar tooth (4, 5, 11, 14).

Based on the evaluation of the fractures in this specific population, several patterns of ramus fractures were identified. Pattern A fractures were more likely to occur following an animal altercation. There was high variability in the fracture lines, but these line were found to lie within specific areas (Figure 2). This information is useful for surgical planning. High fragmentation and displacement scores suggested that non-invasive or minimally invasive techniques are unlikely to provide ideal outcomes. Further research is warranted to develop better treatment options for managing cats with challenging mandibular fractures.

## Data Availability

The original contributions presented in the study are included in the article/supplementary material, further inquiries can be directed to the corresponding author.
